# Supplementary material for: Opening opportunities for Kd determination and screening of MHC peptide complexes
Source: Commun Biol. 2022 May 23;5:488. doi: 10.1038/s42003-022-03366-0 (PMC9127112; doi:10.1038/s42003-022-03366-0)
Supplement: Supplementary file 2 — Supplemental Material [file 42003_2022_3366_MOESM2_ESM.pdf]

SUPPLEMENTARY INFORMATION

Opening opportunities for K_d determination and screening of MHC peptide complexes

Janine-Denise Kopicki[1,2], Ankur Saikia[3], Stephan Niebling[4], Christian Günther[4], Raghavendra Anjanappa[3], Maria Garcia-Alai[4], Sebastian Springer[3]*, Charlotte Uetrecht[1,2,5]*

[1]Centre for Structural Systems Biology (CSSB), Leibniz Institute of Virology (LIV), Notkestraße 85, 22607 Hamburg, Germany.
[2]University of Siegen, Am Eichenhang 50, 57076 Siegen, Germany.
[3] Life Sciences and Chemistry, Jacobs University, Campus Ring 1, 28759 Bremen, Germany.
[4]Centre for Structural Systems Biology (CSSB), European Molecular Biology Laboratory – Hamburg Outstation, Notkestraße 85, Hamburg 22607, Germany.
[5]European XFEL GmbH, Holzkoppel 4, 22869 Schenefeld, Germany

* corresponding authors, email: charlotte.uetrecht@cssb-hamburg.de; s.springer@jacobs-university.de

MHC class I, native MS, peptide binding, peptide affinity, iDSF, antigen presentation, erucamide

THIS PDF FILE INCLUDES:

Figure S1. Small molecule tandem MS analysis verifies that erucamide accounts for a large proportion of the contaminants found in dsA2 samples.

Figure S2. Raw spectrum of dsA2/NV9.

Figure S3. Melting temperature (T_m) of dsA2 in presence of two corresponding truncated NV9 variants.

Figure S4. Overall area under the curve (AUC) for the detected dsA2 mass species at 25 V acceleration voltage.

Figure S5. Overall area under the curve (AUC) for the detected dsA2 mass species at 50 V acceleration voltage.

Table S1. Experimental masses and FWHM for dsA2 and different peptides obtained by native mass spectrometry.

Table S2. Overall area under the curve (AUC) for the detected dsA2 mass species at different acceleration voltages.

Table S3. Apparent dissociation constants (K_d) for dsA2 and different peptides obtained by native MS and iDSF.

Table S4. Melting temperatures (T_m) for dsA2 and different peptides obtained by nDSF.

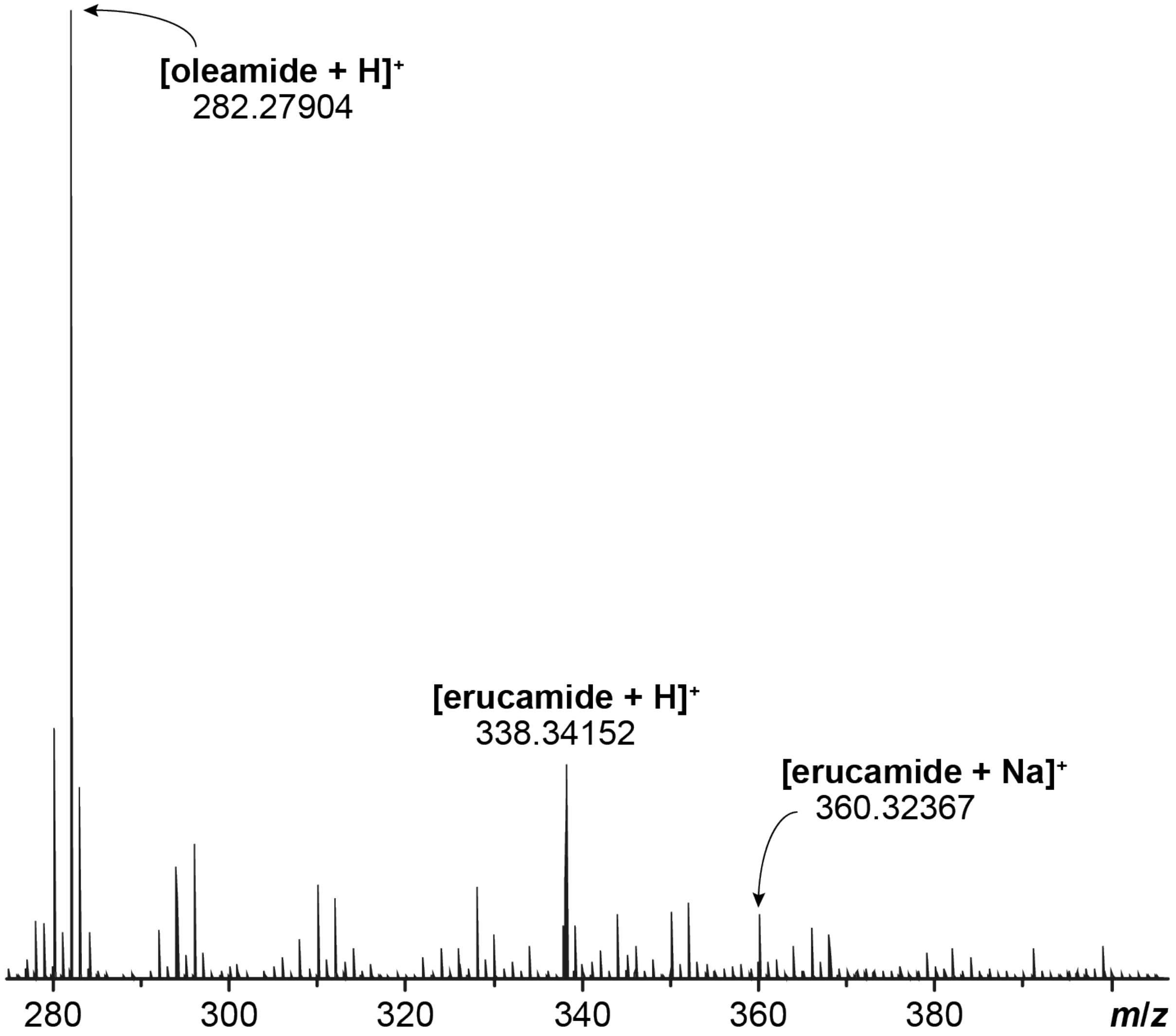

Figure S1. Small molecule tandem MS analysis verifies that erucamide accounts for a large proportion of the contaminants found in dsA2 samples. Displayed is a spectrum resulting from the subtraction of the spectrum of plastic ware contaminants (empty vial) from the one of the dsA2 sample itself. Erucamide (337 Da) is hereby identified as the contaminant adducted to dsA2. Although oleamide is also found in the sample, there was no evidence of binding to the protein in the native MS analysis, unlike for erucamide.

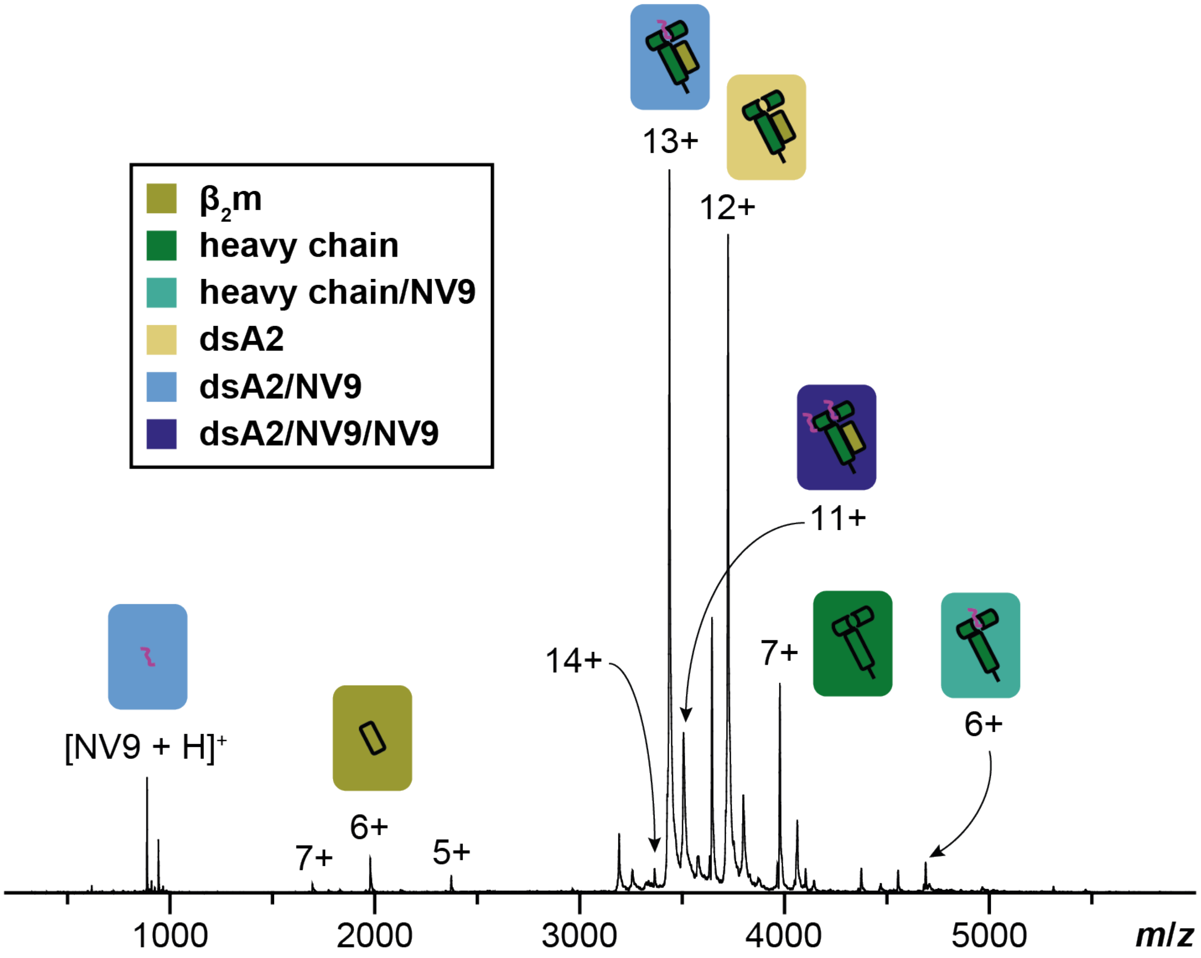

Figure S2. Raw spectrum of dsA2/NV9. A native mass spectrum dsA2 in presence of NV9 (protein-peptide ratio 1:5) recorded at an acceleration voltage of 25 V is shown. The dsA2/NV9 is the predominant species (light blue). In addition, peptide-free dsA2 (yellow), dissociated β_2m (olive) and heavy chain (dark green) as well as free NV9 can be seen.

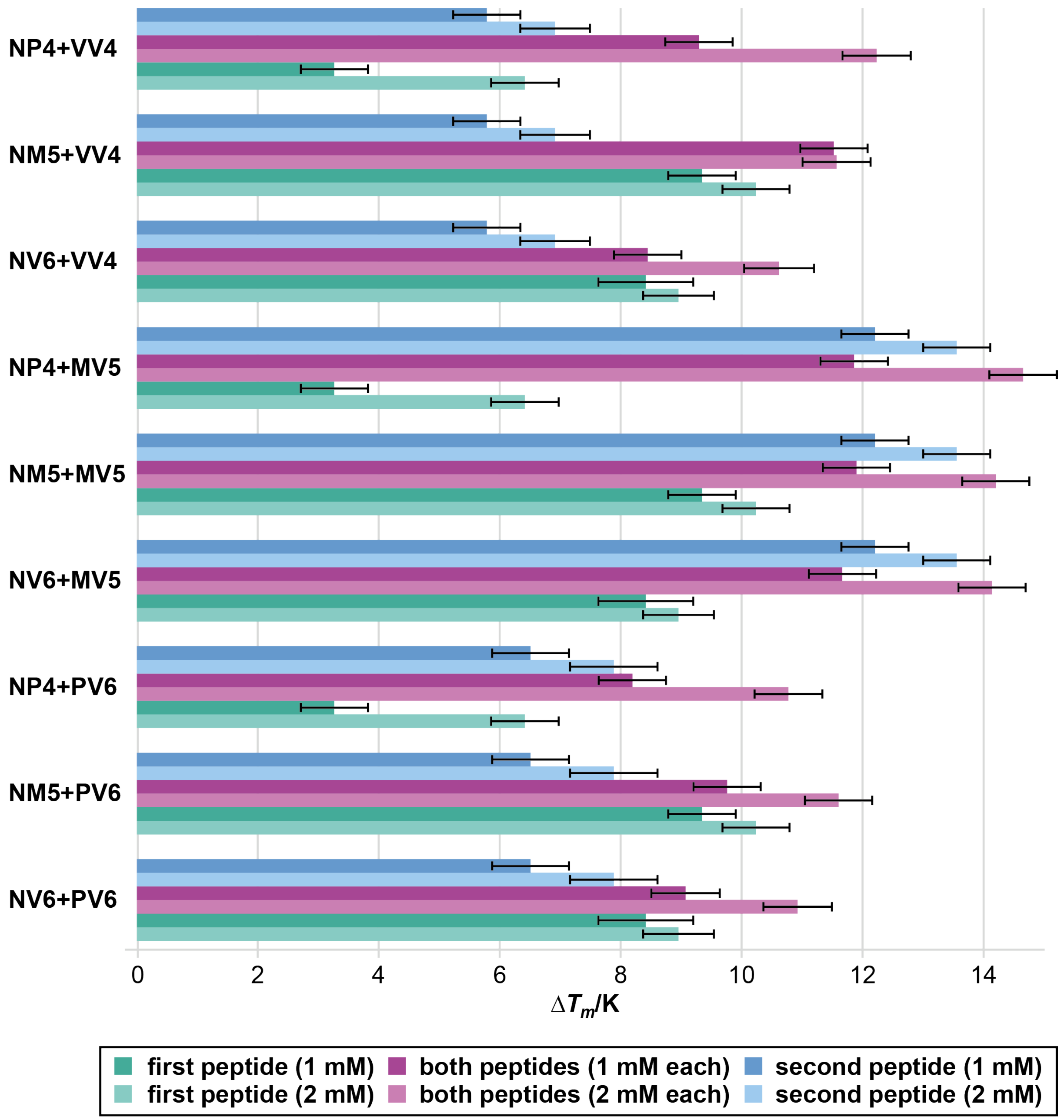

Figure S3. Melting temperature (T_m) of dsA2 in presence of two corresponding truncated NV9 variants. Nanoscale differential scanning fluorimetry is employed to study thermal denaturation of dsA2 in presence of two peptides at once. 2 µM dsA2 are combined with either exclusively the *N*- (teal) or *C*-terminal peptide (blue) or both peptides together (purple) at different concentrations. Results for ΔT_m along with respective standard deviations are displayed.

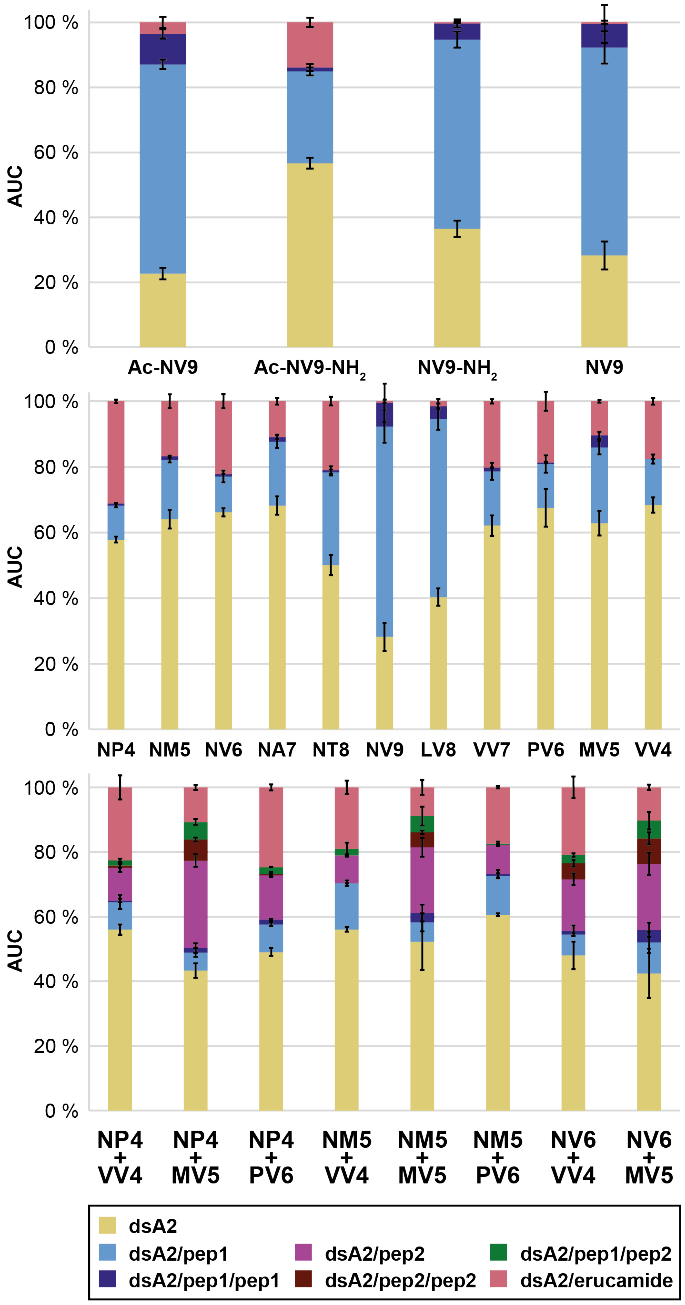

Figure S4. Overall area under the curve (AUC) for the detected dsA2 mass species at 25 V acceleration voltage. The AUC is determined over the entire spectrum for the respective mass species at 25 V. The mean value of the AUC in absence or presence of the different peptides (protein-peptide ratio 1:5, 1:10:10 in dual peptide approach) from at least three independent measurements is depicted along with error bars that represent the corresponding standard deviation. “dsA2” (yellow bars) corresponds to the empty HLA-A*02:01(Y84C/A139C) disulfide mutant complex, “dsA2/pep” (light blue bars) to dsA2 bound to one peptide, “dsA2/pep/pep” to dsA2 bound to two molecules of this certain peptide (dark blue bars), “dsA2/pep2” to dsA2 bound to another peptide when two different peptides where present (purple bars), “dsA2/pep2/pep2” to dsA2 bound to two molecules of the second peptide (dark red bars), “dsA2/pep1/pep2” to dsA2 bound to one molecule of each of both peptides (dark green bars) and “dsA2/erucamide” to dsA2 bound to the erucamide (coral bars) respectively.

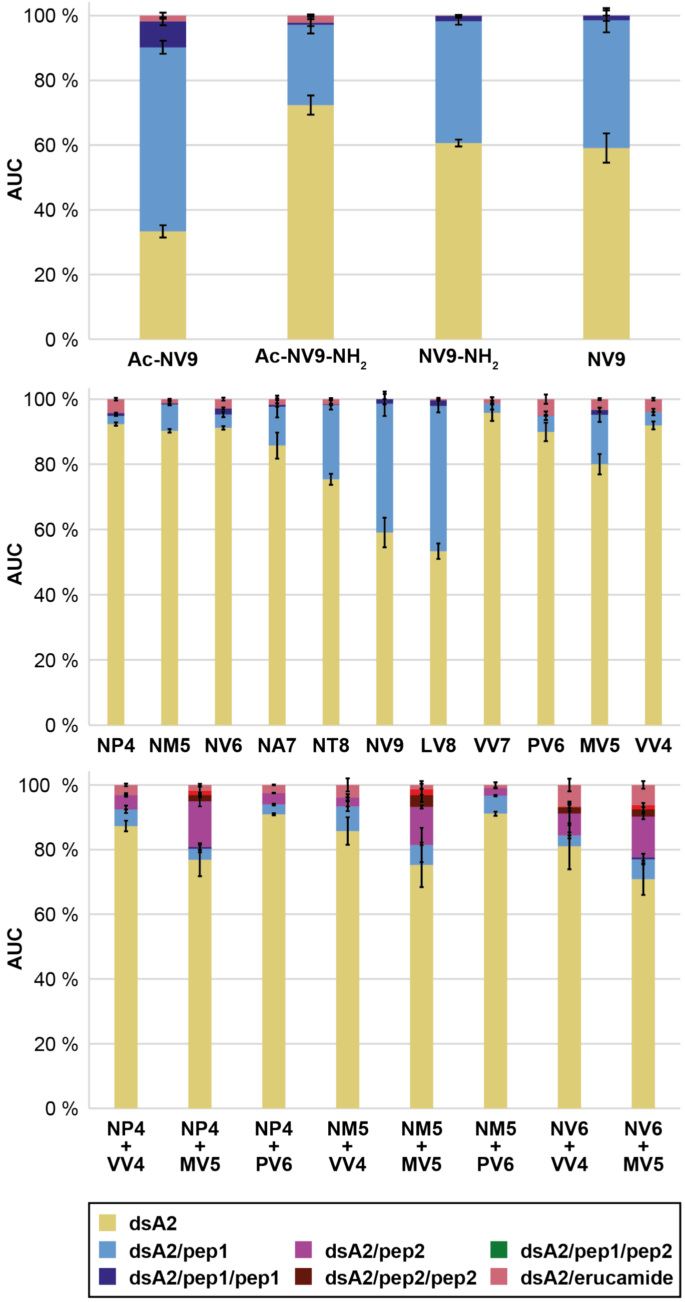

Figure S5. Overall area under the curve (AUC) for the detected dsA2 mass species at 50 V acceleration voltage. The AUC is determined over the entire spectrum for the respective mass species 50 V. The mean value of the AUC in absence or presence of the different peptides (protein-peptide ratio 1:5, 1:10:10 in dual peptide approach) from at least three independent measurements is depicted along with error bars that represent the corresponding standard deviation. “dsA2” (yellow bars) corresponds to the empty HLA-A*02:01(Y84C/A139C) disulfide mutant complex, “dsA2/pep” (light blue bars) to dsA2 bound to one peptide, “dsA2/pep/pep” to dsA2 bound to two molecules of this certain peptide (dark blue bars), “dsA2/pep2” to dsA2 bound to another peptide when two different peptides where present (purple bars), “dsA2/pep2/pep2” to dsA2 bound to two molecules of the second peptide (dark red bars), “dsA2/pep1/pep2” to dsA2 bound to one molecule of each of both peptides (dark green bars) and “dsA2/erucamide” to dsA2 bound to the erucamide (coral bars) respectively.

Table S1. Experimental masses and FWHM for dsA2 and different peptides obtained by native mass spectrometry. Experimental masses (m_{exp}) of the different protein species of disulfide stabilized HLA-A*02:01(Y84C/A139C) disulfide mutant (dsA2) in absence or presence of the different peptides (protein-peptide ratio 1:5, 1:10:10 in dual peptide approach) are determined from at least three independent mass spectrometry measurements. They are listed together with the respective values for standard deviation *s* and average full width of the peak at half maximum (FWHM) along with the theoretically calculated molecular weight (*M*). FWHM values are given for the whole peak area where individual species are not fully resolved. [1]NV9 – high affinity control, [2]YF9 – low affinity control, [3]GV9 – minimal binding motif, [4]Ac-NV9 – modified *N*-terminus, [5]Ac-NV9 NH_2 – modified *N*- and *C*-terminus, [6]NV9-NH_2 – modified *C*-terminus.

mass species	*M*/Da	m_{exp}/Da	$s(m_{exp})$/Da	FWHM/Da	$s(m_{exp})$/Da
dsA2 - M1	43,608	43,603	4	20	20
dsA2	43,739	43,733	4	3	2
heavy chain (hc)	31,877	31,873	2	2.1	0.3
β_2m chain - M1	11,731	11,729	2	1.1	0.4
β_2m chain	11,862	11,860	1	1.4	0.2
dsA2/NV9[1]	44,682	44,678	1	3	2
dsA2/2×NV9	45,625	45,624	4	12	6
hc/NV9	32,820	32,816.9	0.8	2.2	0.3
dsA2/YF9[2]	44,856	44,849	1	7	3
dsA2/GV9[3]	44,369	44,363.8	0.7	2	1
dsA2/2×GV9	44,999	44,995	2	3	2
hc/GV9	32,507	32,503.0	0.5	2.08	0.09
dsA2/Ac-NV9[4]	44,724	44,718.3	0.5	1.6	0.3
dsA2/2×Ac-NV9	45,709	45,704	2	3	2
hc/Ac-NV9	32,862	32,857.3	0.5	1.9	0.1
dsA2/Ac-NV9-NH_2[5]	44,723	44,717.4	1.0	1.4	0.1
dsA2/2×Ac-NV9-NH_2	45,707	45,701.00	1.00	1.8	0.6
hc/Ac-NV9-NH_2	32,861	32,857.0	0.9	1.7	0.2
dsA2/NV9-NH_2[6]	44,681	44,676.4	0.5	1.38	0.06
dsA2/2×NV9-NH_2	45,623	45,619.6	0.9	1.6	0.6
hc/NV9-NH_2	32,819	32,815.1	0.9	1.72	0.10

mass species	M/Da	m_{exp}/Da	$s(m_{exp})$/Da	FWHM/Da	$s(m_{exp})$/Da
dsA2/NP4	44,181	44,176	3	10	10
dsA2/2×NP4	44,623	44,619	4	30	40
hc/NP4	32,319	32,317	2	20	40
dsA2/NM5	44,312	44,309	2	6	3
dsA2/2×NM5	44,885	44,883	2	60	50
hc/NM5	32,450	32,449	1	2.5	0.6
dsA2/NV6	44,411	44411	1	7	2
dsA2/2×NV6	45,083	45,082	3	145	6
hc/NV6	32,549	32,547	3	6	7
dsA2/NA7	44,481	44,476.4	0.5	5	3
dsA2/2×NA7	45,223	45,225	4	30	60
hc/NA7	32,619	32,615	2	2.4	0.5
dsA2/NT8	44,583	44,579.33	1.00	3	2
dsA2/2×NT8	45,427	45,425	2	7	5
hc/NT8	32,721	32,718.1	0.3	2.4	0.7
dsA2/LV8	44,568	44,565.8	0.7	2.6	0.7
dsA2/2×LV8	45,397	45,396.1	0.3	3.4	1.0
hc/LV8	32,706	32,704.8	0.4	2.18	0.04
dsA2/VV7	44,455	44,447.8	0.8	7	3
dsA2/2×VV7	45,171	45,166	3	3	1
hc/VV7	32,593	32,586	3	3	2
dsA2/PV6	44,356	44,350	3	5	3
dsA2/2×PV6	44,973	44969	4	100	50
hc/PV6	32,194	32,487	3	2.3	0.4
dsA2/MV5	44,259	44,254	3	8	12
dsA2/2×MV5	44,779	44,774	4	20	30

mass species	M/Da	m_{exp}/Da	$s(m_{exp})$/Da	FWHM/Da	$s(m_{exp})$/Da
hc/MV5	32,397	32,388.4	1.0	2.1	0.2
dsA2/VV4	44,127	44,125	5	10	10
dsA2/2×VV4	44,515	44,512	8	20	30
hc/VV4	32,265	32,255	1	10	3
dsA2/erucamide	44,077	44,071	5	10	20
dsA2/NP4/VV4	44,569	44,565	2	30	40
dsA2/NP4/MV5	44,701	44,697	2	4	5
dsA2/NP4/PV6	44,798	44,792	3	7	6
dsA2/NM5/VV4	44,700	44,699	2	30	40
dsA2/NM5/MV5	44,832	44,827	2	12	3
dsA2/NM5/PV6	44,929	44,927	2	100	40
dsA2/NV6/VV4	44,799	44,797	4	30	40
dsA2/NV6/MV5	45,451	44927	5	100	60

Table S2. Overall area under the curve (AUC) for the detected dsA2 mass species at different acceleration voltages. The AUC is determined over the entire spectrum for the respective mass species at 10 V, 25 V and 50 V. The mean value of the AUC in absence or presence of the different peptides (protein-peptide ratio 1:5, 1:10:10 in dual peptide approach) from at least three independent measurements is listed together with their corresponding standard deviation *s*. "dsA2" corresponds to the empty HLA-A*02:01(Y84C/A139C) disulfide mutant complex, "dsA2/pep" to dsA2 bound to one peptide, "dsA2/pep/pep" to dsA2 bound to two molecules of this certain peptide, "dsA2/pep2" to dsA2 bound to another peptide when two different peptides where present, "dsA2/pep2/pep2" to dsA2 bound to two molecules of the second peptide, "dsA2/pep1/pep2" to dsA2 bound to one molecule of each of both peptides and "dsA2/erucamide" to dsA2 bound to the erucamide respectively. [1]NV9 – high affinity control, [2]YF9 – low affinity control, [3]GV9 – minimal binding motif, [4]Ac-NV9 – modified *N*-terminus, [5]Ac-NV9 NH2 – modified *N*- and *C*-terminus, [6]NV9-NH2 – modified *C*-terminus.

peptide	acc. volt.	dsA2		dsA2/pep		dsA2/pep/pep		dsA2/pep2		dsA2/pep2/pep2		dsA2/pep1/pep2		dsA2/erucamide	
		AUC	*s*	AUC	*s*	AUC	*s*	AUC	*s*	AUC	*s*	AUC	*s*	AUC	*s*
NV9[1]	10 V	31%	2%	64%	3%	4%	4%							2%	2%
	25 V	28%	4%	64%	5%	7%	6%							0.5%	0.5%
	50 V	59%	5%	40%	4%	1%	2%							0%	0%
YF9[2]	10 V	56%	3%	4%	2%	0%	0%							39%	2%
	25 V	51%	4%	5%	2%	0%	0%							44%	3%
	50 V	95%	5%	5%	2%	0%	0%							2%	2%
GV9[3]	10 V	52%	2%	43%	2%	1.5%	0.4%							4.3%	0.6%
	25 V	50%	1%	43%	3%	2.2%	0.9%							5%	2%
	50 V	62%	3%	35%	3%	1.1%	0.3%							1.7%	0.2%
Ac-NV9[4]	10 V	24%	2%	63%	3%	11%	2%							2.1%	0.5%
	25 V	23%	2%	64%	1%	9%	1%							3%	2%
	50 V	33%	2%	57%	2%	8%	1%							1.7%	0.9%
Ac-NV9-NH2[5]	10 V	55%	2%	27.9%	0.5%	2.4%	0.6%							15%	1%
	25 V	57%	2%	28%	1%	1%	1%							14%	1%
	50 V	72%	3%	25%	3%	1%	1%							2.2%	0.4%
NV9-NH2[6]	10 V	36%	2%	59%	2%	5%	1%							0.4%	0.2%
	25 V	36%	2%	58%	2%	5%	1%							0.3%	0.3%
	50 V	61%	1%	38%	1%	1.6%	0.3%							0.1%	0.2%

peptide	acc. volt.	dsA2		dsA2/pep		dsA2/pep/pep		dsA2/pep2		dsA2/pep2/pep2		dsA2/pep1/pep2		dsA2/erucamide	
		AUC	*s*	AUC	*s*	AUC	*s*	AUC	*s*	AUC	*s*	AUC	*s*	AUC	*s*
NP4	10 V	55%	2%	11%	1%	0.6%	0.2%							33.8%	0.6%
	25 V	57.8%	0.9%	10.4%	0.5%	0.69%	0.06%							31.1%	0.5%
	50 V	92.4%	0.5%	2.6%	0.1%	0.8%	0.1%							4.3%	0.3%
NM5	10 V	62%	2%	17.9%	0.6%	1.0%	0.2%							19%	1%
	25 V	64%	3%	18.0%	0.7%	1.2%	0.2%							17%	2%
	50 V	90.3%	0.6%	8.1%	0.3%	0.3%	0.6%							1.3%	0.1%
NV6	10 V	66%	1%	10%	2%	0.5%	0.1%							24%	1%
	25 V	66%	1%	11%	2%	0.69%	0.08%							22%	2%
	50 V	91.2%	0,5%	4.1%	0.8%	1.9%	0.3%							2.9%	0.4%
NA7	10 V	75%	3%	16%	2%	0.8%	0.3%							8%	1%
	25 V	68%	3%	19%	2%	1.4%	0.7%							11%	1%
	50 V	86%	4%	12%	3%	0.5%	0.5%							1.8%	0.2%
NT8	10 V	47.3%	0.9%	28%	1%	0.6%	0.5%							24%	1%
	25 V	50%	3%	28.2%	0.9%	1%	1%							21%	1%
	50 V	75%	2%	23%	1%	0.2%	0.3%							1.6%	0.3%
LV8	10 V	42%	6%	54%	5%	2.8%	0.3%							1.7%	0.9%
	25 V	40%	3%	54%	3%	3.9%	0.8%							1.5%	0.7%
	50 V	53%	2%	45%	2%	1.6%	0.2%							0.4%	0.4%
VV7	10 V	61.8%	0.5%	17%	2%	1.7%	0.3%							19%	3%
	25 V	62%	3%	17%	3%	1.16%	0.06%							20.2%	0.7%
	50 V	96%	3%	3%	2%	0%	0%							1.3%	0.6%
PV6	10 V	68%	6%	12%	2%	0.5%	0.6%							19%	3%
	25 V	68%	6%	13%	3%	0.5%	0.2%							19%	3%
	50 V	90%	3%	5%	1%	0.1%	0.1%							5%	1%

peptide	acc. volt.	dsA2		dsA2/pep		dsA2/pep/pep		dsA2/pep2		dsA2/pep2/pep2		dsA2/pep1/pep2		dsA2/erucamide	
		AUC	s	AUC	s	AUC	s	AUC	s	AUC	s	AUC	s	AUC	s
MV5	10 V	64%	3%	23%	2%	3.2%	0.7%							10.4%	0.2%
	25 V	63%	4%	23%	2%	3.7%	1.0%							10.4%	0.4%
	50 V	80%	3%	15%	2%	1.5%	0.6%							3.3%	0.3%
VV4	10 V	68%	1%	14%	1%	0%	0%							18.093%	0.007%
	25 V	68%	2%	14%	1%	0%	0%							17.6%	1.0%
	50 V	92%	1%	4.1%	1.0%	0%	0%							4.0%	0.4%
NP4 + VV4	10 V	54%	2%	9%	3%	1.1%	0.5%	10%	1%	1.2%	1.0%	2.1%	0.5%	22%	4%
	25 V	56%	2%	8%	2%	0.5%	0.5%	10%	1%	0.7%	0.7%	1.6%	0.5%	22%	4%
	50 V	87%	2%	5%	1%	0%	0%	4.4%	0.6%	0%	0%	0%	0%	3.1%	0.4%
NP4 + MV5	10 V	42.9%	0.9%	6%	2%	1,8%	0.3%	27%	1%	5.6%	0.3%	5.2%	0.8%	10%	1%
	25 V	43%	2%	5%	1%	1%	2%	27%	2%	6.4%	0.6%	5.3%	0.9%	10.5%	0.8%
	50 V	76%	5%	3%	1%	0.5%	0.9%	14%	2%	1.9%	0.8%	1%	1%	1.8%	0.3%
NP4 + PV6	10 V	48%	3%	8%	1%	1.7%	0.3%	13%	2%	0.9%	0.8%	2.8%	0.2%	25%	2%
	25 V	49%	1%	8.5%	0.6%	1.4%	0.2%	13.6%	0.5%	0.4%	0.7%	2.1%	0.1%	24.5%	0.9%
	50 V	90.8%	0.3%	3.0%	0.3%	0%	0%	3.5%	0.1%	0%	0%	0%	0%	2.5%	0.1%
NM5 + VV4	10 V	56%	3%	13%	2%	1%	1%	8.9%	0.8%	0.5%	0.8%	1.8%	1.6%	18%	4%
	25 V	55.7%	0.7%	14.2%	0.9%	0%	0%	8.6%	0.4%	0%	0%	2.0%	1.9%	19%	2%
	50 V	86%	4%	8%	1%	0%	0%	2.8%	1.0%	0%	0%	0%	0%	4%	2%
NM5 + MV5	10 V	46%	7%	8%	3%	5%	4%	20%	1%	4.9%	0.8%	6%	1%	9%	1%
	25 V	51%	9%	6%	3%	3%	3%	20%	3%	4.5%	0.5%	5%	3%	9%	2%
	50 V	75%	7%	6%	5%	0%	0%	11.7%	0.4%	4%	2%	2%	2%	1%	1%
NM5 + PV6	10 V	58%	2%	11.9%	0.6%	1%	2%	9%	2%	0%	0%	0.4%	0.7%	18%	1%
	25 V	60.3%	0.5%	12.0%	0.8%	1%	1%	8.9%	0.4%	0%	0%	0.4%	0.7%	17.3%	0.4%
	50 V	91.1%	0.7%	5.6%	0.2%	0%	0%	2.4%	0.2%	0%	0%	0%	0%	0.9%	0.8%

peptide	acc. volt.	dsA2		dsA2/pep		dsA2/pep/pep		dsA2/pep2		dsA2/pep2/pep2		dsA2/pep1/pep2		dsA2/erucamide	
		**AUC**	***s***	**AUC**	***s***	**AUC**	***s***	**AUC**	***s***	**AUC**	***s***	**AUC**	***s***	**AUC**	***s***
NV6 + VV4	10 V	47%	8%	6%	1%	1%	0%	17%	4%	7%	4%	3%	0%	18%	1%
	25 V	48%	4%	6%	0%	1%	2%	16%	2%	5%	1%	2%	1%	21%	3%
	50 V	81%	7%	3%	1%	0%	0%	7%	4%	2%	1%	0%	0%	7%	2%
NV6 + MV5	10 V	47%	5%	8%	2%	4%	2%	21%	1%	6.5%	0.8%	3.0%	0.3%	10%	2%
	25 V	42%	8%	9%	3%	4%	2%	20%	3%	8%	2%	5%	3%	10.2%	0.8%
	50 V	71%	5%	6%	2%	1%	1%	12.5%	0.9%	2.1%	0.7%	1.4%	0.6%	6%	1%

Table S3. Apparent dissociation constants (K_d) for dsA2 and different peptides obtained by native MS and iDSF. The K_d is calculated from the respective area under the curve values at 10 V acceleration voltage (protein-peptide ratio 1:5). $K_{d,high}$ is an affinity determined on the basis of a real experiment at a cone voltage of 150 V, while a theoretical cone voltage of 36 V is assumed for $K_{d,low}$ to correct for ion-source decay. $K_{d,iDSF}$ is derived by two independent measurements. Protein concentration is 2.2 µM. For each ligand, a two-fold serial dilution series is prepared using 11 concentrations depending on their predicted or assumed K_d range. The listed standard deviation *s* for K_d is determined using common equations, which estimate the propagation of uncertainty. [1]NV9 – high affinity control, [2]GV9 – minimal binding motif, [3]Ac-NV9 – modified *N*-terminus, [4]Ac-NV9 NH2 – modified *N*- and *C*-terminus, [5]NV9-NH2 – modified *C*-terminus; *iDSF reaches its limits at affinities below 200 nm, hence the values of grayed-out peptides are not reliable.

peptide	sequence	$K_{d,high}$		$K_{d,low}$		$K_{d,iDSF}$	
		K_d/µM	*s*/µM	K_d/µM	*s*/µM	K_d/µM	*s*/µM
NV9[1]	NLVPMVATV	8	2	0.06	0.08	0.04*	0.01
GV9[2]	GLGGGGGGV	35	2	0.5	0.2	0.36	0.06
Ac-NV9[3]		4.5	1.0	0.11	0.05	0.61	0.08
Ac-NV9-NH2[4]		80	7	7	3	4	1
NV9-NH2[5]		10	1	0.004	0.003	0.001*	0.001
NP4	NLVP	350	60	100	70	50	20
NM5	NLVPM	180	30	15	6	11	3
NV6	NLVPMV	380	70	30	10	9	2
NA7	NLVPMVA	210	40	2.0	0.7	3.6	0.5
NT8	NLVPMVAT	92	5	30	10	2.6	0.4
LV8	LVPMVATV	17	3	0.07	0.06	0.008*	0.005
VV7	VPMVATV	180	30	15	7	7.8	1.0
PV6	PMVATV	300	200	15	7	15	2
MV5	MVATV	110	10	3	1	1.6	0.2
VV4	VATV	270	30	13	4	50	20
NP4+VV4				90	30		
NP4+MV5				11	3		
NP4+PV6				130	50		
NM5+VV4				50	20		
NM5+MV5				9	3		

peptide	sequence	$K_{d,high}$		$K_{d,low}$		$K_{d,iDSF}$	
		K_d/µM	s/µM	K_d/µM	s/µM	K_d/µM	s/µM
NM5+PV6				50	10		
NV6+VV4				50	10		
NV6+MV5				13	4		

Table S4. Melting temperatures (T_m) for dsA2 and different peptides obtained by nDSF. The T_m as well as the resulting s for dsA2 in absence or presence of the different peptides is defined by at least two independent measurements. Protein concentration is 2 µM. [1]NV9 – high affinity control, [2]YF9 – low affinity control, [3]GV9 – minimal binding motif, [4]Ac-NV9 – modified *N*-terminus, [5]Ac-NV9 NH2 – modified *N*- and *C*-terminus, [6]NV9-NH2 – modified *C*-terminus.

peptide	0.2 µM		2 µM		20 µM		1 mM		2 mM	
	T_m/°C	s/°C	T_m/°C	s/°C	T_m/°C	s/°C	T_m/°C	s/°C	T_m/°C	s/°C
empty	35.7	0.6								
NV9[1]	35.8	0.8	58.96	0.07	59.1	0.1	60.698	0.007		
YF9[2]	36.1	0.8	36.1	0.8	36.2	0.8				
GV9[3]	36.4	0.6	38.8	0.4	42.1	0.2	47.6	0.1		
Ac-NV9[4]	36.6	0.7	39.6	0.6	43.2	0.6	48.420	0.001		
Ac-NV9-NH2[5]	36.0	0.8	36.6	0.4	38.4	0.3				
NV9-NH2[6]	36.0	0.9	46.9	0.2	50.3	0.3	55.53	0.04		
NP4	35.6	0.4	35.7	0.5	35.8	0.4	38.95	0.04	42.10	0.06
NM5	35.6	0.5	35.9	0.4	37.3	0.3	45.04	0.07	45.93	0.03
NV6	35.5	0.1	35.9	0.2	37.61	0.05	44.1	0.6	44.6	0.2
NA7	35.51	0.09	35.9	0.1	37.95	0.06	45.442	0.007		
NT8	35.5	0.1	36.02	0.09	38.78	0.02	46.89	0.02		
LV8	35.40	0.08	43.5	0.1	47.28	0.04	53.30	0.04		
VV7	35.5	0.1	35.7	0.2	37.0	0.1	42.29	0.01		
PV6	35.5	0.2	35.62	0.09	36.40	0.02	42.2	0.3	43.6	0.5
MV5	35.64	0.08	36.5	0.1	39.31	0.07	47.90	0.01	49.253	0.003
VV4	35.475	0.002	35.1	0.6	35.5	0.2	41.47	0.03	42.6	0.1
NP4+VV4							44.99	0.05	47.93	0.10
NP4+MV5							47.55	0.06	50.35	0.05
NP4+PV6							43.88	0.05	46.47	0.08
NM5+VV4							47.22	0.05	47.26	0.08
NM5+MV5							47.59	0.03	49.900	0.002
NM5+PV6							45.45	0.02	47.295	0.010

peptide	0.2 μM		2 μM		20 μM		1 mM		2 mM	
	***T**m*/°C	***s***/°C	***T**m*/°C	***s***/°C	***T**m*/°C	***s***/°C	***T**m*/°C	***s***/°C	***T**m*/°C	***s***/°C
NV6+VV4							44.14	0.05	46.3	0.2
NV6+MV5							47.36	0.04	49.84	0.02
NV6+PV6							44.8	0.1	46.6	0.1
